# Allergic March in Children: The Significance of Precision Allergy Molecular Diagnosis (PAMD@) in Predicting Atopy Development and Planning Allergen-Specific Immunotherapy

**DOI:** 10.3390/nu15040978

**Published:** 2023-02-15

**Authors:** Izabela Knyziak-Mędrzycka, Emilia Majsiak, Bożena Cukrowska

**Affiliations:** 1 Outpatient Allergology Clinic, The Children’s Memorial Health Institute, Aleja Dzieci Polskich 20, 04-730 Warsaw, Poland; 2 Department of Health Promotion, Chair of Nursing Development, Faculty Health of Sciences, Medical University of Lublin, Staszica 4/6, 20-081 Lublin, Poland; 3 Department of Pathomorphology, The Children’s Memorial Health Institute, Aleja Dzieci Polskich 20, 04-730 Warsaw, Poland

**Keywords:** precision allergy molecular diagnostic applications, PAMD@, allergic march, molecular spreading, allergen-specific immunotherapy

## Abstract

The allergic march is a progression of naturally occurring symptoms whose nature changes with age. The classic allergic march typically begins in infancy and manifests in the form of atopic dermatitis and food allergy. As immune tolerance develops over time, these conditions may resolve by the age of 3–5 years; however, they may evolve into allergic rhinitis and bronchial asthma. Traditional diagnostic assessments, such as skin prick testing or serum allergen-specific immunoglobulin E (sIgE) level testing, are conducted to introduce effective treatment. Recent years saw the emergence of precision allergy molecular diagnosis (PAMD@), which assesses sIgE against allergenic molecules. This new technology helps more accurately evaluate the patient’s allergy profile, which helps create more precise dietary specifications and personalize allergen-specific immunotherapy. This review presents possible predictions regarding the allergic march and the means of controlling it based on PAMD@ results.

## 1. Introduction

Allergy that occurs in the first years of a child’s life is typically food allergy (FA) [1]. Due to its increasing incidence, FA is an important problem in clinical pediatrics. FA may manifest with mild symptoms, such as abdominal discomfort, nausea, vomiting, or diarrhea, but also with severe, life-threatening symptoms, which may be due to immunoglobulin E (IgE)-mediated hypersensitivity (e.g., anaphylactic shock) (Table 1) or due to severe dehydration and electrolyte imbalance resulting from intense vomiting or diarrhea caused by non-IgE-mediated reactions [2,3]. Hypersensitivity to foods is often also the first step in the so-called allergic march—a progression from FA to inhalant allergy, which leads to asthma [4]. Usually, one of the first FA manifestations is atopic dermatitis (AD), which is a common condition in early childhood, with an estimated prevalence of 15–25% in children [5]. Data from the literature show that approximately 45% of infants develop AD symptoms before the age of 6 months, with the proportion rising to 50% by the age of 1 year. Comorbid IgE-mediated FA and AD in infancy and early childhood are the earliest manifestations of the atopic march [6,7]. Symptomatic FA, especially severe or multiple ones, was shown to be closely associated with bronchial asthma in children aged ≥6 years [8]. Children with FA developed bronchial asthma earlier than children without this allergy [9]. Another study revealed that milk sensitivity in infancy predisposes the child to severe respiratory tract infections and airway hypersensitivity to histamine [10]. The authors of a pediatric cohort study [11] assessing the progression of symptoms in the allergic march observed a systematic relationship between FA in infancy and inhalant allergies at the age of 3–6 years.

There are a number of literature reports on wide-scope community-based and dietary interventions in an attempt to prevent the allergic march, to most effectively limit allergic inflammation, and to create barriers against allergic march progression at an early age [12,13,14,15]. Other allergy prevention strategies, ranging from dietary interventions to therapeutic procedures, are secondary in nature. They involve allergen-specific immunotherapy, which is instrumental in symptom management when the allergic condition has already developed [16].

The need for new management strategies drives researchers to look for biomarkers that could help identify the causative factors of food hypersensitivity. Identifying specific protein allergens responsible for hypersensitivity reactions helps estimate the risk of allergic symptom progression and the effectiveness of planned therapies. Precision allergy molecular diagnosis (PAMD@), which involves evaluating specific IgE (sIgE) against allergenic molecules, is a state-of-the-art technique available in everyday allergy practice.

Of course, it should be emphasized that the mere presence of sIgE in relation to allergen molecules, as in the case of allergen extracts, is not sufficient to diagnose allergies. The presence of sIgE antibody detected with the use of extracts or molecules in in vivo or in vitro assays is only an indicator of allergic sensitization and not allergic disease itself.

Undoubtedly, comparing the known benefits it brings, PAMD@ will prove useful both in primary and secondary prevention of childhood allergies.

The purpose of this review was to discuss the significance of PAMD@ in predicting atopy development and planning effective allergen-specific immunotherapy in the pediatric population.

**Table 1:** The most common food allergens involved in allergic manifestations in infants (based on [17]).

Species	Allergenic Molecule	Protein Family Name	Sensitization Rate in Specific Species (%)	Resistance to Heating and Chemical Denaturation	Allergic Symptoms ^2^
**Cow milk**, *Bos domesticus*	Bos d 4	α-lactalbumin	51	Moderate	Oral allergy syndrome (OAS), abdominal pain, bloating, nausea, diarrheaskin changes, atopic dermatitis, asthma, edema, allergic rhinitisallergic conjunctivitis, anaphylactic shock
	Bos d 5	β-lactoglobulin	61	Low	
	Bos d 6	Serum albumin	43	Low	
	Bos d 8	Casein	63	High	
**Eggs**, *Gallus domesticus*	Gal d 1	Ovomucoid		High	
	Gal d 2	Ovoalbumin		Low	
	Gal d 3	Ovotrasferrin		Low	
	Gal d 4	Lysozyme		Moderate	
**Fish**, e.g., *Gadus callarias*	Gad c 1	Parvalbumin	100	High	
**Shellfish**, e.g., *Penaeus monodan*	Pen m 1	Tropomyosin	62	High	
**Nuts**, e.g., *Corylus avellana*	Cor a 1	Bet v 1-like	90	Low	
	Cor a 8	11S globulin	36–83	High	
	Cor a 9	nsLTP ^1^	5.8	High	
**Peanuts**, *Arachis hypogaea*	Ara h 1	Vicilin	63–80	High	
	Ara h 2	2S albumin	90	High	
	Ara h 3	Legumin		High	
	Ara h 6	2S albumin	76–96	High	
	Ara h 8	Bet v 1-like		Low	
	Ara h 9	nsLTP ^1^		High	
**Soy**, *Glycine max*	Gly m 4	Bet v 1-like	10.3	Low	
	Gly m 5	Vicilin	33	High	
	Gly m 6	Legumin		High	
	Gly m 8	2S albumin		High	
**Wheat**, *Triticum aestivum*	Tri a 14	nsLTP		High	
	Tri a 19	Omega-5-gliadin	50–70	High	

^1^ nsLTP—Non-specific lipid-transfer protein. ^2^ Not all of these symptoms may occur with all molecules listed, and other symptoms not listed here may also occur. The presence of sIgE for some molecules (e.g., Ara h 1, Ara h 2) is associated with a higher risk of severe reactions.

## 2. PAMD@ Assays

The means of determining the cause of allergy in routine laboratory diagnostics involve measuring the levels of sIgE against the most common allergens (including allergens of foods, such as cow’s milk, eggs, wheat, soy, nuts, and fish, and inhalant allergens, such as birch, timothy grass, house dust mites, molds, and animal allergens) [17]. Developed several years ago, PAMD@ is a state-of-the-art form of allergy diagnostics, which helps establish the allergy type (primary/cross-reaction), course (depending on the type of protein allergens), and prognosis (transient/persistent allergy) [17,18]. The use of PAMD@ makes it possible to identify individual allergen molecules and assess them comprehensively via multiplex testing. This approach seems more appropriate if we stop thinking about egg, milk, and other foods as allergens and begin to construe these foods as a source of many different allergens, as shown in Table 1.

Singleplex PAMD@ is used for assessing the serum levels of sIgE against individual allergenic molecules. Depending on the technical characteristics (solid-phase and liquid-phase assays, various solid-phase substrates, native and recombinant components, different types of detection antibodies, and different types of enzyme interactions), the tests have varied sensitivity and specificity. Singleplex tests yield quantitative results but require the use of relatively large serum sample volumes (40–50 µL of serum per allergen, plus the so-called dead space volume of approximately 100 µL), and their cost per single assay is relatively high [19]. Conversely, multiplex PAMD@ involves the simultaneous determination of sIgE for multiple allergen components in a single assay. The first multiplex assay was ImmunoCAP ISAC, capable of analyzing sIgE against a total of 112 allergen components from 50 allergen sources. This was followed by the emergence of third-generation nanotechnology applications. The first of such assays was FABER (which is now no longer produced), capable of simultaneously analyzing 122 molecules and 122 allergen extracts, and another was ALEX (after changes in 2019—ALEX2), capable of analyzing 178 allergen molecules and 117 allergen extracts. One unquestionable advantage of ALEX2 tests over other assays is the presence of a cross-reactive carbohydrate determinant (CCD) inhibitor, which greatly reduces false-positive results. Moreover, unlike the ImmunoCAP ISAC assay, which is semi-quantitative, ALEX2 is a quantitative assay [17].

## 3. Allergy Prognosis Based on PAMD@

Since the introduction of PAMD@ several years ago, the usefulness of this technique in predicting the course of disease has been increasingly emphasized. Earlier assays to determine the sIgE against allergen extracts, which had been in use since the 1960s, offered only a rough overview of the patient’s allergy status. Recent years brought the possibility of determining the patient’s precise allergy profile, thanks to the introduction of PAMD@.

### 3.1. PAMD@, Allergy Symptoms, and Provocation Testing

PAMD@ seems to be useful in predicting the type and severity of allergic symptoms. In the case of cow’s milk allergy, determining the sIgE to individual allergen components helps identify patients allergic to casein (Bos d 8), who are at a high risk for anaphylactic reactions, and those allergic to alpha-lactalbumin (Bos d 4) or beta-lactoglobulin (Bos d 5), whose risk of severe anaphylaxis is lower and who can be expected to develop milder symptoms, mainly in the form of skin lesions or gastroenteritis [17]. Another example is egg allergy, where the detection of sIgE against ovomucoid (Gal d 1), which is an egg protein, is associated with high risk of anaphylaxis [17]. PAMD@ can also be used in predicting the results of allergen provocation tests. Depending on the type of test used, the levels of casein-specific IgE that have been reported to be predictive of a positive oral cow’s milk provocation test range from 0.95 kU/L to 10.0 kU/L [20,21,22,23]. Ando et al. reported Gal d 1-specific IgE levels of over 4.4 kU/L to be associated with positive egg provocation challenge results in children [24]. Conversely, other egg-derived molecules, such as ovalbumin (Gal d 2) and ovotransferrin (Gal d 3) very rarely produce a positive provocation test result [25]. 

Moreover, PAMD@ helps predict if the allergy is temporary or persistent. In a prospective study, Dang et al. determined the levels of sIgE to egg molecules (Gal d 1, 2, 3, and 5) and to an egg protein extract in three subgroups of 12-month-old infants [25]. These subgroups were infants with egg white allergy confirmed via allergen provocation testing, infants with egg sensitivity, and those with egg tolerance. The study was followed up at the ages of 2 and 4 years and showed that Gal d 1 sensitization increased the risk of long-term egg allergy five-fold, and the presence of sIgE to all egg allergens (Gal d 1, 2, 3, and 5) increased the risk of persistent allergy to raw eggs four-fold. Moreover, the authors demonstrated that egg allergy in infants increased the risk of aeroallergen sensitization and was associated with an increased risk of developing a respiratory allergy (asthma, rhinitis) by the age of 4 years. An earlier study presented that an egg allergy in infancy, particularly if it coexisted with AD, increased the risk of developing respiratory symptoms and hypersensitivity to aeroallergens in early childhood [26]. 

Similarly, studies on peanut allergy have demonstrated a relationship between the severity and type of allergy and symptom progression. A study by Sicherer et al. conducted on 511 children showed that the cut-off sIgE levels associated with clinically significant peanut allergy were ≥5 kU/L in children aged ≤2 years and ≥14 kU/L in those older than 2 years. The authors concluded that high-risk groups include non-breastfed infants and infants with high Ara h 2-specific IgE levels [27]. A systematic review and meta-analysis involving component-resolved diagnostics of peanut allergy indicated that the use of PAMD@ can lead to establishing the final diagnosis in a more rapid and safer way while reducing the number of unnecessary oral allergen provocation tests with peanut allergens [28].

### 3.2. Assessment of Molecular Spreading

PAMD@ also helps us observe the phenomenon of molecular spreading, which involves progressive sensitization to other allergenic molecules from a given source in patients initially sensitized only to a single type of allergenic molecules. This phenomenon was described by Matricardi et al. who evaluated the course of sensitization to timothy grass (*Phleum pratense*) in a boy from the age of 3 to 10 years. Initially, at the age of 3 years, the boy was diagnosed only with sensitivity to Phl p 1 [29]. Subsequently, at the age of 6, he was also found to have Phl p 2 sensitivity. By the age of 10 years, the boy had become sensitized to Phl p 4, Phl p 5, Phl p 6, and Phl p 11 molecules. The authors of that study hypothesized that the introduction of allergen-specific immunotherapy would stop or inhibit this molecular spreading and the associated progression of allergy symptoms [29]. Posa et al. followed up with pediatric patients to evaluate the extent of their allergy by analyzing the sIgE against molecules of *Dermatophagoides pteronyssinus* over a period of 20 years [30]. The most common molecules (>40%) detected early in their lives were Der p 2, Der p 1, and Der p 23 (molecules of group A), followed by (15–30%) Der p 5, Der p 7, Der p 4, and Der p 21 (molecules of group B). The least common (<10%) sensitivities were to Der p 11, Der p 18, Der p 16, Der p 14, and Der p 15 (molecules of group C). Sensitization usually started with group A proteins. Over time, blood tests revealed the presence of sIgE to group B allergens and, eventually, to group C molecules. Early-onset sensitization, extensive exposure to house dust mites, and parental allergic rhinitis were associated with the development of overtly symptomatic allergy during the subsequent years. The patients sensitized to all house dust mite allergen groups listed above (groups A + B + C) were at a significantly higher risk of allergic rhinitis and bronchial asthma. The presence of serum sIgE against Der p 1 or Der p 23 at the age of 5 years or younger was a positive prognostic factor for the development of asthma by school age [30]. In the case of birch allergy, Westman et al. showed a correlation between the risk of emerging or persistent symptoms of allergic rhinitis in the evaluated children at the age of 16 and the presence of Bet v 1-specific IgE at the age of 4 years [31]. Moreover, they demonstrated that high Bet v 1-specific IgE levels at the age of 4 were associated with severe allergic rhinitis at the age of 16 years. Other evaluated PR10 proteins showed a hierarchic correlation: Bet v 1 > Mal d 1 > Cor a 1.04 > Ara h 8 > Pru p 1 > Aln g 1 > Api g 1 > Act d 8 > Gly m 4. The proteins from the PR10 group that were recognized by sIgE were also associated with the risk of developing allergic rhinitis and oral allergy syndrome [31].

## 4. PAMD@ and Allergen-Specific Immunotherapy

The data verified based on the PAMD@ results can be invaluable in preparing a patient for immunotherapy [32]. Such data help personalize the immunotherapy vaccine, which improves the effectiveness of the entire course of immunotherapy and increases the chances for successful management of atopy [33]. Diagnosing allergy based on allergenic molecules also helps assess if the patient’s hypersensitivity is associated with the so-called true allergy or cross-reactivity, which may be very important in making decisions on causative treatment.

Until recently, the selection of vaccines for allergen-specific immunotherapy was very challenging in patients with both symptomatic allergic rhinitis in the season from March to June and the presence of sIgE against birch and timothy allergen extracts [34]. Currently, PAMD@ helps determine whether the result obtained via conventional techniques is due to cross-reactivity between pollen allergens or to true allergy. Selecting the appropriate allergen, the one that causes the allergy, determines the achieved clinical response and the effectiveness of immunotherapy. 

The content of whole-allergen extracts is highly variable. They may not even contain clinically significant allergen components, which may pose diagnostic difficulties and lead to suboptimal immunotherapy vaccine selection. Frick et al. evaluated Api m 1 and Api m 10 levels in commercially available whole-allergen extracts used for bee venom immunotherapy [35]. The absence of Api m 10 in the extract (found in 3 out of 5 analyzed samples) was associated with a ten-fold higher risk of immunotherapy failure. Determining the Api m 10 levels and, possibly, the use of an allergen extract containing Api m 10 are indicated in clinical practice in patients who have failed to respond to immunotherapy, which is supposed to protect the patient against an anaphylactic reaction to a bee sting [35].

It is also possible for a patient to present with obvious allergy symptoms but have undetectable serum sIgE levels or negative skin prick test results. Nonetheless, once individual allergen molecules are analyzed, the final diagnosis may be sensitivity to a molecule that is completely absent from standard whole-allergen extracts or whose levels in those extracts are very low. This may be due to the methods used to manufacture the test extract or to the physicochemical properties of the given allergen [36], which can be exemplified by Api m 10 components (which play an important role in bee venom immunotherapy) or Der p 10 and Der p 23 components (which play a role in house dust mite allergy) [37]. Casset et al. analyzed *D. pteronyssimus* allergen extracts from 10 different manufacturers. Only the Der p 1 and Der p 2 components were detected in all analyzed extracts. However, even then, their levels and relative proportions varied significantly. Moreover, at least one of the four expected molecules (Der p 5, 7, 10, and 21) was not detectable in 8 out of the 10 analyzed allergen extracts. Importantly, the lack of Der p 10 (tropomyosin) in diagnostic extracts makes it difficult to diagnose potentially clinically significant cross-reactivity allergy to shrimp and other seafood. The authors of that study concluded that the available house dust mite allergen extracts vary greatly in their allergen content and may not contain clinically significant allergen components, which may lead to diagnostic problems [38]. It is worth noting that the molecular makeup of both immunotherapy vaccines and whole-allergen extracts is similar [39]. Therefore, immunotherapy should not be initiated in patients who test negative for major house dust mite allergens (Der p 1, Der p 2, Der p 23, Der f 1, and Der f 2) [17].

## 5. The Lower Limit of Normal sIgE Levels in PAMD@

The discovery of IgE by Ishizaka and by Johansson and Bennich reported in 1960 was an important milestone in allergy diagnosis [40]. Another important milestone was a method of detecting the serum levels of IgE against individual allergens. Sensitivity and specificity analysis of the diagnostic tests available in the 1970s helped establish the lower limit of normal sIgE levels to be 0.35 kU/L [41]. The issue of lowering this lower limit below 0.35 kU/L has been recently suggested and discussed [42]. Nilsson et al. demonstrated that food allergen-specific IgE levels of 0.1–0.34 kU/L (in the case of allergens such as eggs, milk, or nuts) detected in infancy increase the risk of developing inhalant allergies at the age of 5 years, and—in the case of low levels of egg-specific IgE—also the risk of developing AD in early childhood [43]. Therefore, sIgE levels in the first year of life, despite being below 0.35 kU/L, may be an additional predictive factor of allergy development. Recently, our research team described a case of a 9-month-old boy with an anaphylactic reaction following the intake of cow’s milk and eggs. His blood tests showed high levels of sIgE against milk and egg components and Bet v 1-specific IgE levels below 0.35 kU/L [44]. The boy’s Bet v 1-specific IgE levels measured at 26 months were low at 0.27 kU/L, whereas those measured at 37 months were 2.33 kU/L. Clinical manifestations of allergy, whose timing was consistent with the birch pollen season, occurred already at sIgE levels below 0.35 kU/L. There have also been studies conducted in adult patients that have shown the significance of low-allergen molecule-specific IgE levels (with a range of 0.1–0.34 kU/L) in diagnosing allergies. Balsells-Vives et al. demonstrated that although most patients with symptoms of peach allergy had Pru p 3-specific IgE levels of ≥0.35 kU/L, nearly 50% of the peach allergy patients had low Pru p 3-specific IgE levels of 0.1–0.34 kU/L [45].

Clinically evident allergic disease appears to be present when the IgE antibody level is greater than 0.35 kU/L, but not always, and it depends highly on the specific-to-total IgE ratio, the total IgE of the patient, the extent of allergen exposure, and the “sensitivity” of the patient’s mast cells. Nevertheless, the interpretation of sIgE results in the range of 0.1 to 0.35 kU/L should be made with caution.

IgE is a marker of sensitization but not allergic disease. It is only one of many “risk” factors for the development of an allergic reaction. IgE antibody levels above 0.35 kUa/L have been associated with a higher probability or a greater risk of clinical allergic disease manifestations. However, allergic disease cannot be determined by the test itself but rather by the physician who takes into consideration the patient’s clinical history of type 1 hypersensitivity-associated reactions following a relevant allergen exposure and other patient and environmental risk factors (e.g., IgE antibody levels, total serum IgE levels, the patient’s family genetic history, the severity of the reported reactions, type of reactions, possible cross-reactive allergen exposure).

## 6. PAMD@ Not for Everyone

Currently, PAMD@ is not meant to be part of routine allergy diagnostics. Disease-management protocols, including those on patient eligibility to undergo immunotherapy, still require positive allergen extract-based sIgE or positive skin prick test results [46]. Nonetheless, this method serves the additional function of helping to make the results more precise. PAMD@ is not intended for monosensitized patients with a predictable, seasonal pattern of allergy symptoms. These patients require only routine diagnostic tests involving sIgE levels or skin prick testing in order to receive immunotherapy.

With the development and greater availability of PAMD@, practical questions of clinicians are: “how to” and “when” to use molecular allergen diagnosis and whether such a diagnostic strategy is appropriate in terms of costs and predictive values. There is no single optimal answer to these questions. Each case should be considered individually based on the clinical condition of the patient. Nevertheless, we can identify certain groups of patients who benefit most from the use of PAMD@ in the process of diagnosing their clinical symptoms. These are subjects classified for immunotherapy (patients allergic to single or several inhalant allergens, patients with multiple allergies to pollen or with allergy to *Hymenoptera insects*), patients with anaphylaxis (after food, with the participation of cofactors, with delayed anaphylaxis after red meat, idiopathic anaphylaxis), patients with latex allergy, with polysensitization (especially those with a co-existence of sensitization to inhalant and food allergens), and patients with food allergy (to assess the risk of the severity of allergic reactions and to identify unexpected sources of sensitization). At this point, the importance of clinicians should also be emphasized. They are responsible for deciding when and which diagnostic strategy should be used, taking into account the patient’s symptoms and the local law.

## 7. Limitations of PAMD@

One of the limitations is the number of molecules marked with PAMD@. Currently, over a thousand molecules are described, but we can routinely label fewer than 200 of them (Table 2). Due to cost constraints and the clinical value of the results, only very few component sIgE antibodies are routinely run on singleplex assays today. For example, peanut molecules (Ara h 1, Ara h 2, Ara h 3, Ara h 8, and Ara h 9) are routinely performed due to their established value in confirming risk for mild versus severe reactions and cross-reactivity with the PR10 and nsLTP allergen families. However, it should be emphasized that we can now determine sIgE against many clinically important molecules, but more studies are necessary to prove their usefulness in clinical practice. On the other hand, it should be noted that in some cases, e.g., cow’s milk allergy, the determination of sIgE against allergen components does not bring greater benefits than the determination of sIge based on allergen extracts. The improper interpretation of sIgE results based on allergen extracts or individual molecules may lead to the overdiagnosis of the patient and unnecessary elimination of a given product from the patient’s diet, which may result in a loss of tolerance to a given product. Therefore, sIgE testing should be ordered and interpreted very carefully by the clinicians based on the patient’s clinical symptoms.

Another limitation is that some important molecules cannot be determined otherwise than by means of multiplexes [17,47]. One example of such proteins is oleosins, which have been shown to be important in patients who have a history of anaphylaxis after consuming peanuts, sunflower seeds, or soy and who have skin or blood tests negative for allergen extracts. The determination of proteins from this group allows for the assessment of the risk of severe anaphylaxis and the clarification of ambiguous cases of allergy [47]. 

As in the case of allergen extract-based diagnostics, the results of PAMD@ may not always be consistent with clinical manifestations. One of the possible explanations for this phenomenon involves the CCDs that are present in some proteins [17,48]. Anti-CCD antibodies may produce positive results in in vitro allergy tests, which may hinder the clinical interpretation of laboratory test results. This problem may affect up to 30% of patients. The source of the CCDs that in turn activate CCD-specific IgE synthesis are usually plant pollen allergens and insect venoms [19,48]. 

We have naturally glycosylated and non-glycosylated molecules and allergen extracts. Clinicians’ knowledge of molecular structure seems to be of great importance in interpreting the effect of anti-CCD on sIgE results from allergen molecules and extracts. Currently, we have several approaches to limit the impact of anti-CCD on sIgE results for allergens, such as: (1) the use of recombinant allergen molecules (if it does not reduce the diagnostic sensitivity for molecules); (2) the determination of sIgE against CCDs in the patients’ sera; (3) the use of a blocker against anti-CCDs (as an additional reagent or routinely in a standard procedure). Of course, individual solutions have their advantages and disadvantages. For example, the use of CCD inhibitors or recombinant proteins in sIgE testing is not a good solution for all molecules. The use of deglycosylated forms of Tri aA_TI and Api g 5 molecules has been shown to reduce the sensitivity of wheat allergen-specific and celery allergen-specific IgE tests, respectively [49,50]. In addition, in case of doubt, in vivo skin tests can always be performed or a provocation test can be carried out. Obtaining the knowledge and experience necessary for interpreting PAMD@ test results may require a certain amount of dedication [17]. Education on PAMD@ seems to be necessary to help clinicians obtain the knowledge required for interpreting the results.

## 8. Costs of PAMD@ in Allergy Diagnosis

It is also worth emphasizing the cost of PAMD@ use in diagnosing allergies. Singleplex and multiplex PAMD@ tests (Table 2) are relatively expensive, and this is why they are not used routinely. However, we can look at the cost of a multiplex test in terms of the number of results it provides. A multiplex test result provides more than a hundred individual test results, thanks to which we can obtain a personalized detailed allergic profile of the patient. This allows us to look at the cost of using PAMD@ in allergy diagnostics from a different perspective.

Recent reports have underlined the potential of PAMD@ in the field of health economics and have suggested that it can help to save costs for the diagnosis of allergies [51,52]. The detailed sensitization profile also improves the effectiveness of immunotherapy. Although immunotherapy is still clinically based, it is PAMD@ that allows for a more precise selection of specific immunotherapy vaccines, which can reduce the cost [53].

We would like to especially highlight the role of multiplex tests to reduce costs. More than a hundred individual test results from one sample can present a comprehensive picture of allergen sensitization, which can be used for precise medical treatment. In fact, unrecognized and untreated allergy is a major cost factor for the management of allergic diseases [54,55,56]. Early detection of the disease and the implementation of an elimination diet can not only reduce the costs of diagnostic processes and subsequent therapy but can also significantly improve the quality of life of patients, as we have demonstrated in our research on autoimmune diseases [57,58]. With adherence to proper treatment and the precise diagnosis and proper management of allergic diseases, it has been estimated that even high costs can be saved [54,55,56].

## 9. Summary

Using PAMD@ in allergy diagnostics and reducing the lower limit of normal sIgE levels are intended to diagnose allergies as early as possible and help assess the risk of molecular spreading and anaphylaxis. PAMD@ is also a state-of-the-art tool that helps to make decisions on the introduction of causative treatment—allergen-specific immunotherapy and personalized selection of immunotherapy vaccines. The actions taken based on the information obtained via PAMD@ may help stem allergy development. Considering the substantial usefulness of PAMD@ in the clinical management of patients with inconclusive results of routine allergy tests, having this technology at our disposal makes allergic march control seem more achievable.

## Figures and Tables

**Table 2:** Characteristics of selected tests used for PAMD@.

Test	Manufacturer	The Technic of Determination	Type of Designation	Type of Test	Number of Molecules Possible to be Determined ^3^	Lower Limit of Detection
ImmunoCap^®^	Thermo Fisher Scientific Inc., Waltham, MA, USA	Fluorescence enzyme immunoassay	Quantitative	Singleplex	100	0.1 kU/L
Polycheck^®^	Biocheck GmbH, Münster,	Solid-phase immunoassays	Quantitative	Multiparametric	34	0.15 kU/L
Euroline^®^	Euroimmun AG, Lübeck, Germany	Solid-phase immunoassays	Semiquantitative	Multiparametric	35	0.35 kU/L
ImmunoCap^®^ ISAC	Thermo Fisher Scientific Inc., Waltham, MA, USA	Solid-phase immunoassay	Semiquantitative	Multiplex	112	0.35 ISU-E ^1^
ALEX^®^2	MacroArray Diagnostics, Vienna, Austria	Solid-phase immunoassay	Quantitative	Multiplex	178 ^2^	0.1 kU/L

^1^ ISU-E—Standardized units for specific IgE. ^2^ In addition to allergen molecules, the ALEX^®^2 test also determines the sIgE to 117 extracts and the total IgE. ^3^ Information on the number of molecules comes from the official catalogs of the test manufacturers (or distributors) from their official websites.

## Data Availability

The data presented in this study are available on request from the corresponding author.
